# Quality of prescribing and health-related quality of life in older adults: a narrative review with a special focus on patients with atrial fibrillation and multimorbidity

**DOI:** 10.1007/s41999-025-01175-2

**Published:** 2025-03-09

**Authors:** Cheima Amrouch, Deirdre A. Lane, Amaia Calderón-Larrañaga, Mirko Petrovic, Delphine De Smedt, the AFFIRMO investigators

**Affiliations:** 1 Department of Internal Medicine and Paediatrics, Ghent University, Ghent, Belgium; 2 Department of Public Health and Primary Care, Ghent University, Ghent, Belgium; 3 Liverpool Centre for Cardiovascular Science, University of Liverpool, Liverpool John Moores University and Liverpool Heart and Chest Hospital, William Henry Duncan Building, Liverpool, L7 8TX UK; 4 Department of Cardiovascular and Metabolic Medicine, Institute of Life Course and Medical Sciences, University of Liverpool, Liverpool, UK; 5 Department of Clinical Medicine, Aalborg University, Aalborg, Denmark; 6 Department of Neurobiology, Aging Research Center, Care Sciences and Society, Karolinska Institutet and Stockholm University, Stockholm, Sweden; 7 Stockholm Gerontology Research Center, Stockholm, Sweden

**Keywords:** Quality of prescribing, Health-related quality of life, Atrial fibrillation, Potentially inappropriate prescribing, Multimorbidity, Older adults

## Abstract

**Purpose:**

To summarise the association between potentially inappropriate prescribing (PIP) and health-related quality of life (HRQOL) in older adults, with a special focus on those with atrial fibrillation (AF) and multimorbidity, while exploring potential interventions to improve prescribing quality and their impact on HRQOL.

**Methods:**

A comprehensive search strategy was conducted in MEDLINE using the PubMed interface on August 16th, 2024, focusing on key terms related to “potentially inappropriate prescribing” and “quality of life”. Additionally, the reference lists of included studies were screened. Only studies utilising validated assessment tools for HRQOL or measuring global self-perceived health status were considered. Studies involving populations with an average age of  ≥ 65 years were included.

**Results:**

Of the 1810 articles screened, 35 studies were included. The findings indicate that the quality of prescribing, independent of polypharmacy, may negatively influence HRQOL. The review identified a range of interventions aimed at improving prescribing quality among older adults, including pharmacist-driven, general practitioner-driven, and multidisciplinary approaches. Interventions were assessed among distinct population groups and specifically in residential care homes. While some interventions demonstrated improvements in prescribing quality, the overall evidence regarding their impact on HRQOL remains limited.

**Conclusion:**

The relationship between prescribing quality and HRQOL remains underexplored in older adults with AF and multimorbidity, despite the high prevalence of PIP. Effective pharmacotherapy should be coupled with a comprehensive assessment of patients' clinical and functional parameters, considering their HRQOL. Adopting a multidisciplinary, integrated, patient-centred approach is essential for sustainable and appropriate prescribing practices and may enhance HRQOL.

**Supplementary Information:**

The online version contains supplementary material available at 10.1007/s41999-025-01175-2.

## Key summary points

**Aim:**

What is the relationship between quality of prescribing and health-related quality of life in older adults with atrial fibrillation and multimorbidity, and what interventions can improve both?

**Findings:**

The findings suggest a statistically significant association between PIP and lower health-related quality of life. The review identified various interventions aimed at improving quality of prescribing, including pharmacist-driven, general practitioner-driven, and multidisciplinary approaches. Although some interventions showed improvements in quality of prescribing, the evidence regarding their impact on health-related quality of life remains limited, especially in older adults with atrial fibrillation and multimorbidity.

**Message:**

There is a critical need for well-designed studies to address potentially inappropriate prescribing and explore the impact of integrated patient-centred interventions on HRQOL in older adults with atrial fibrillation and multimorbidity.

**Supplementary Information:**

The online version contains supplementary material available at 10.1007/s41999-025-01175-2.

## Introduction

When a cure is not readily available, as is often the case for older adults with chronic conditions, the primary focus of healthcare shifts to symptom management and preventing disease progression. In this context, measuring health-related quality of life (HRQOL) should serve as an indicator of treatment effectiveness, taking into consideration an individual’s expectations and preferences [1–3].

Although there is no universal consensus on the definition of HRQOL [4], this review defines it as the impact of a condition, its symptoms, and treatment on a person’s self-perceived health status and well-being [4, 5]. HRQOL is a subjective and individualised measure influenced by various factors, including demographic, psychological, and socioeconomic characteristics [6]. In accordance with the biopsychosocial model, HRQOL is a dynamic and multidimensional concept, encompassing physical, social, and psychological aspects of well-being and functioning [7].

HRQOL is a relevant patient-reported outcome measure for older adults with multiple chronic conditions [7, 8]. Older adults with atrial fibrillation (AF) often present with multimorbidity, where the most prevalent comorbidities include hypertension, heart failure, coronary artery disease, diabetes mellitus, and obstructive sleep apnoea [9]. Polypharmacy, common in those with multimorbidity, complicates medication management and increases the risk of potentially inappropriate prescribing (PIP), an umbrella term for over-, under- and misprescribing [10]. PIP can be categorised as potentially inappropriate medications (PIMs) or potential prescribing omissions (PPOs). PIMs are medications that pose a greater risk than benefit to the patient, or medications that are prescribed with inappropriate dosages, frequencies, routes of administration, durations, or without a clear indication (diagnosis) [10]. PIP has been associated with adverse patient-related outcomes and impaired HRQOL [11, 12]. The quality of prescribing can be determined by identifying PIP.

The need for an integrated approach to AF management has been previously highlighted [13]. The currently ongoing H2020 AFFIRMO project aims to develop and evaluate a holistic, patient-centred care strategy based on the ABC model [14]. The ABC pathway—focusing on (A) stroke prevention, (B) better symptom control, and (C) comorbidity management—has shown promise [15–17]. A registry-based study suggested that adherence to the ABC model is associated with better HRQOL [18]. While factors such as symptom severity, age, sex and multimorbidity have been studied in relation to HRQOL in older adults with AF, the impact of polypharmacy and the quality of prescribing on HRQOL has received less investigation [12, 19, 20].

The present review summarises the association between the quality of prescribing and HRQOL in the general population and explores potential interventions that may improve prescribing quality and hence its possible impact on HRQOL. Understanding these links is essential for developing strategies to optimise prescribing and consequently improving patient-related outcomes, also in older adults with AF and multimorbidity.

## Methodological considerations

### Search strategy and eligibility criteria

A search of the published peer-reviewed literature was conducted on August 16th, 2024. MEDLINE, accessed via the PubMed interface, was searched using key terms related to "potentially inappropriate prescribing" and "quality of life" (Online Resource 2). Reference lists of included studies were also screened. No restrictions were placed on publication year, language, or study design. However, conference abstracts, editorials, and case reports were excluded, due to their lower evidence quality and limited information. Studies involving populations with an average age of ≥ 65 years and those utilising validated assessment tools for HRQOL or measuring global self-perceived health status were considered. Validated assessment tools refer to measurement instruments that have been tested for their reliability and accuracy in assessing HRQOL or self-perceived health status. Additionally, studies assessing (i) the association between PIP and HRQOL, or (ii) the impact of prescribing interventions on HRQOL were included. Screening was performed by one reviewer (C.A.), resulting in 1810 initial results and a final selection of 35 studies. Some original studies included in this review are also covered in the cited systematic reviews. In such cases, priority was given to the systematic reviews to provide a broader synthesis of the evidence. If an original study offered additional insights not captured in the systematic review, the original study was cited.

### Health-related quality of life assessment tools

Several tools have been developed to assess HRQOL, which can be broadly categorised into generic and specific tools [3, 21]. General health perception is often measured with a single global question, asking individuals to rate their health on a scale from poor to excellent [3]. Generic tools have wide applications across different populations and diseases, allowing for comparison between different conditions and the performance of cost-effectiveness studies by generating a utility score to quantify a patient's overall health state (Online Resource 3, table S1) [22]. However, a limitation of these tools is that they are sometimes not sensitive enough to detect specific changes in HRQOL. In contrast, specific tools are designed to focus on aspects directly related to the primary area of interest [21]. These tools may be specific to a disease (Online Resource 3, table S1), dimension, population or medication enabling more precise identification of changes in HRQOL, though they do not permit comparisons across the primary area of interest [21, 23]. Several studies have utilised broader QOL tools to measure HRQOL in older adults [24] to capture aspects directly related to HRQOL, alongside other dimensions such as enjoyment, stability, and social relations.

### Potentially inappropriate prescribing assessment tools

To address PIP, clinical guidelines and various explicit and implicit tools have been developed (Online Resource 3, table S2). Explicit tools use predefined criteria to identify PIP in specific clinical situations, while implicit tools consist of quality indicators and rely on the professional judgement of healthcare providers, focusing on the patient’s overall clinical profile rather than solely their medications and diseases [25].

### Polypharmacy and health-related quality of life

Studies across various populations, including community-dwelling older adults and those with specific health conditions, demonstrate an association between polypharmacy and reduced HRQOL (Table 1). In community-dwelling older adults (≥ 75 years, *n* = 579), polypharmacy was linked to lower HRQOL, as measured by the EuroQol-5 Dimension-5 Level questionnaire (EQ-5D-5L) and the EQ visual analogue scale (EQ-VAS), even after adjusting for age, sex, comorbidities, and cognitive function [26]. Similar findings were observed in older adults with type II diabetes [27].

**Table 1:** Association between polypharmacy and health-related quality of life (HRQOL)

Author, year	Country	Definition polypharmacy	Tools to assess HRQOL	Study design	Study population	Association polypharmacy and HRQOL	DOI
Al-Musawe 2020	Portugal	≥ 5	EQ-5D-3L, EQ-VAS cut-off value 0.60	Cross-sectional	Older adults with type 2 diabetes aged ≥ 65 years (*n* = 670)	OR 1.80 95% CI [1.15; 2.82]	https://doi.org/10.1002%2Fprp2.621
Gallagher 2020	United Kingdom	4–6 polypharmacy > 7 hyperpolypharmacy	EQ-5D and SF-12	Systematic review (1 study assessed HRQOL)	Older adults with AF (*n* = 1762)	EQ5D: parameter estimate ± SE (− 0.06 ± 0.03) SF-12: PCS score (− 3.75 ± 1.03), no significant association with the MCS score	http://doi.org/10.1136/openhrt-2020-001257
González-González 2021	Netherlands and Germany	≥ 5	EQ-5D-3L poor HRQOL defined as decrease of ≥ 5% from baseline	Prognostic model	General practice patients with multimorbidity (≥ 2) and polypharmacy (≥ 5), (*n* = 3582, 78 ± 7 years)	Polypharmacy was not a significant predictor	https://doi.org/10.1016/j.jclinepi.2020.10.006
Piccoliori 2021	Italy	8–9 polypharmacy ≥ 10 hyperpolypharmacy	EQ-5D-5L, EQ-VAS	Cross-sectional	Community-dwelling general practice patients aged ≥ 75 years with polypharmacy (*n* = 579)	Hyperpolypharmacy compared to polypharmacy: OR 0.87 *p* = 0.008 *95%CI not reported* *Adjusted for age, sex, number of conditions, cognitive impairment*	https://doi.org/10.1186/s12877-021-02141-w
Kochar 2024	United States	≥ 5 concomitant medications	PROBE cut-off < 24	Cross-sectional	Adults ≥ 60 years with inflammatory bowel disease (*n* = 100)	OR 22.79 *p* < 0.01, *95% CI not reported* *Adjusted for age, sex, IBD-type, number of comorbidities and depression*	https://doi.org/10.1007/s10620-023-08250-3

*EQ* EuroQol, *SF* Short‐Form Health Survey, *PROBE* Patient-Reported Outcome Based Evaluation of Quality of Life, *OR* odds ratio, *CI* confidence interval, *SE* standard error, *PCS* Physical component summary, *MCS* Mental component summary, *IBD* inflammatory bowel disease

While most studies used generic tools like the EQ-5D and 12‐item Short‐Form Health Survey (SF-12), one study employed a disease-specific tool, the Patient-Reported Outcome-Based Evaluation, in older adults with inflammatory bowel disease. This tool focuses solely on evaluating the psychosocial components of HRQOL, and it was found that polypharmacy was associated with lower HRQOL [28]. A recent systematic review identified only one study examining polypharmacy and HRQOL in older adults with AF and reported that taking seven or more medications was significantly associated with lower overall EQ-5D. No detailed analysis of the individual EQ-5D dimensions was provided. However, when using the SF-12 tool, a significant association was observed only with the physical component [29].

The issue of confounding by indication is particularly relevant when examining the impact of polypharmacy on HRQOL in older adults with multimorbidity. An association between disease burden and HRQOL is well-established; however, distinguishing the specific contributions of treatment burden (polypharmacy) versus disease burden to HRQOL remains challenging. This difficulty arises because polypharmacy and disease burden are strongly correlated. Additionally, the baseline health status of patients with multimorbidity, may further complicate this distinction, as pre-existing conditions affect HRQOL, and the impact might differ depending on the nature of the conditions [20].

A prognostic model to predict deteriorating HRQOL in older adults with multimorbidity, was developed using the EQ-5D-3L measure. The results indicated that polypharmacy was not associated with HRQOL. Instead, potentially inappropriate medications, medication omissions, age, functional status, and baseline HRQOL were identified as significant predictors, with functional status and baseline HRQOL being the most influential factors [30]. The focus should, therefore, be on the appropriateness of pharmacotherapy, rather than the number of medications, as judicious polypharmacy can improve health outcomes and consequently ameliorate HRQOL.

### Quality of prescribing and health-related quality of life

Studies examining the association between the quality of prescribing and HRQOL have yielded mixed results. Both the quality of prescribing and HRQOL were assessed using a range of different tools for each measure (Table 2). In inpatients (*n* = 5 studies) [31, 32], and primary care populations (*n* = 3 studies) [11], some studies (*n* = 6) found that PIP was associated with lower HRQOL, while others (*n* = 2) report no significant relationship [11, 31, 32]. In community-dwelling older adults, exposure to two or more PIPs was associated with a lower HRQOL utility score, with an adjusted coefficient of − 0.09 (SE 0.02) [33].

**Table 2:** Association between quality of prescribing and health-related quality of life (HRQOL)

Author, year	Country	Tool to assess quality of prescribing	Tool to assess HRQOL	Study design	Study population	Association quality of prescribing and HRQOL	DOI
Cahir 2014	Ireland	STOPP/STARTv1	EQ-5D	Cohort study, 6 m FU	Community dwelling adults ≥ 70 years (*n* = 931)	Exposure to one PIP (adjusted coefficient − 0.01, SE 0.02) and to ≥ 2 PIP (adjusted coefficient − 0.09, SE 0.02) associated with lower HRQOL utility *Adjusted for sex, age, social class, Charlson comorbidity index, number of drug classes, self-reported adherence, patients’ perceived level of social support and social network*	https://doi.org/10.1111/bcp.12161
Meid 2016	Germany	STARTv2	EQ-5D, EQ-VAS, SF-12	Population-based cohort study, 11 years FU	Ambulatory patients (*n* = 989), *(73.2* ± *6.1 years)*	Increase in number of omitted drugs by one drug associated with 1.29 point 95% CI [− 2.40;− 0.19] lower HRQOL (EQ-VAS). Similar trend observed in EQ-5D scale of the model-based recursive partitioning *Adjusted for sex, age, cognitive status, weight, GP consultations, CIRS-G-Score, change in perceived impairment by prior unresolved diagnostic questions, current physical disorders, current unresolved diagnostic questions*	https://doi.org/10.1007/s00228-016-2047-8
Moriarty 2016	Ireland	STOPP/STARTv1	CASP-12	Prospective cohort study, 2 years FU	Community dwelling older adults aged ≥ 65 years (*n* = 1753)	Exposure to ≥ 2 potential prescribing omissions was associated with reduced HRQOL (β = − 1.05 95% CI [− 1.83;− 0.26] *Adjusted for age group, sex, level of educational attainment, living arrangements, number of repeat medicines and number of doctor-diagnosed chronic conditions*	https://doi.org/10.1111/bcp.12995
Wallace 2017	Ireland	STOPPv1 Beers 2012	EQ-5D-3L	Prospective cohort study, 2 years FU	Community dwelling older adults ≥ 70 years (*n* = 904)	Exposure to ≥ 2 PIMs identified with STOPP was associated with poorer HRQOL (adjusted coefficient:− 0.11 95% CI [− 0.16;− 0.06]) *Adjusted for age, sex, deprivation, education, social class, number of medications, comorbidity, medication adherence, vulnerability, social support and depression*	https://doi.org/10.1093/gerona/glw140
Akkawi 2019	Malaysia	STOPP/STARTv2	EQ-5D-3L, EQ-VAS	Cross-sectional	Hospitalised older adults ≥ 65 years (*n* = 502)	No statistically significant differences were observed between PIP and the EQ-5D dimensions or EQ-VAS	https://doi.org/10.1007/s11136-019-02153-5
Byrne 2019	Ireland	Drug Burden Index	CASP-19	Cohort study	Community dwelling older adults aged ≥ 65 years (*n* = 1946)	Drug burden index score ≥ 1 reduced HRQOL (β = − 1.84 95%CI [− 3.14;− 0.54]) *Adjusted for polypharmacy, age, sex, education, living arrangements, number of chronic diseases, depression and cognitive function*	https://doi.org/10.1186/s12877-019-1138-7
Liew 2019	Ireland	STOPP/STARTv1, Beers 2012	EQ-5D, CASP-12	Systematic review with meta-analysis of observational studies	Older adults from primary care ≥ 65 years	Pooled analysis: (RR –0.26 95% CI [− 0.36; − 0.16])	https://doi.org/10.1370/afm.2373
Moriarty 2019	Ireland	STOPPv1, Beers 2012, ACOVEv3, STARTv1	CASP-12	Prospective cohort study, 2 years FU	Community dwelling older adults aged ≥ 65 years (*n* = 1753)	Exposure to ≥ 2 potential prescribing omissions (START) was associated with reduced HRQOL (adjusted mean difference = − 1.12 95% CI [− 1.92; − 0.33]) *Adjusted for depressive symptoms, social participation, age, sex, number of medicine, number of diseases, education, living arrangement, cognitive function*	https://doi.org/10.1111/jgs.16239
Al-Musawe 2020	Portugal	STOPP/STARTv2	EQ-5D-3L, EQ-VAS, cut-off value 0.60	Cross-sectional	Older adults with type 2 diabetes (*n* = 670) *(73.01* ± *6.22)*	PIMs (STOPP) (OR 1.57 95% CI [1.0; 2.28])	https://doi.org/10.1002%2Fprp2.621
Saqlaine 2020	Pakistan	Beers 2015	EQ5D-3L, EQ-VAS	Cross-sectional	Older cardiac outpatients ≥ 65 years (*n* = 386)	PIMs associated with 4 EQ-5D dimensions: mobility (*χ*^2^ = 7.53, *p* = 0.006), self-care (*χ*^2^ = 13.38, *p* = 0.001), usual activities (*χ*^2^ = 7.67, p = 0.006), and pain/discomfort (*χ*^2^ = 6.39, *p* = 0.011). No statistically significant association with depression Increasing number of PIMs significantly associated with lower EQ-5D (β = − 0.040 95% CI [− 0.075;− 0.005]) and EQ-VAS (β = − 1.69 95% CI [− 2.916; − 0.456]) *Adjusted for age, sex, family structure, education, annual income, employment status, residence, self-reported health, marital status, hospital visits, hospital admission, fall history, activities of daily livings, comorbidities*	https://doi.org/10.1007/s11136-020-02530-5
González-González 2021	Netherlands and Germany	STOPP/STARTv2	EQ-5D-3L, poor HRQOL defined as decrease of ≥ 5% from baseline	Prognostic model	General practice patients with multimorbidity (≥ 2) and polypharmacy (≥ 5) (*n* = 3582), *(78* ± *7 years)*	PIMs (estimate ± SE (1.108 ± 0.432)) and potential prescribing omissions (0.386 ± 0.159) statistically significant predictors of HRQOL	https://doi.org/10.1016/j.jclinepi.2020.10.006
Mekonnen 2021	Sweden, Denmark, Malaysia, Switzerland	STOPPv1, STOPP/STARTv2, Red-Yellow-Green list, MAI	EQ-5D, EQ-VAS	Systematic review (4 studies assessed HRQOL)	Inpatient hospital setting *(age range 72.4–83.4 years)*	Two studies found a significant association between PIMs, identified with STOPPv2, and Red-Yellow-Green list, and HRQOL. One study assessing quality of prescribing with MAI also identified an association between lower medication quality and lower HRQOL	https://doi.org/10.1111/bcp.14870
Hughes 2023	Ireland	British National Formulary, Stockley's Drug Interactions	EQ-5D-5L	Cross-sectional	Older adults (≥ 65 years) acutely hospitalised (*n* = 798)	EQ-5D was statistically significantly lower in those exposed to ≥ 1 DDI (0.49 ± 0.39) compared to those without DDI (0.57 ± 0.41), *p* = .03	https://doi.org/10.1111/bcp.15970
Clark 2024	United States	Beers 2019	SF-12	Cross-sectional	Community dwelling older adults aged ≥ 65 years (*n* = 218,383,123)	PIMs was associated with poorer PCS scores across all age groups with those ≥ 85 years (adjusted regression coefficient = − 1.65 95% CI [− 3.03;− 0.27]). A statistically significant association was also found with MCS scores for age groups 65–74 years and ≥ 85 years (adjusted regression coefficient in those ≥ 85 years = − 2.01 95% CI [− 3.25;− 0.78]) *Adjusted for age, sex, race, marital status, education, income, insurance coverage, ADL limitations, IADL limitations, geographic region, coronary heart disease, angina, myocardial infarction, heart failure, chronic renal failure, cancer, arthritis, hypertension, dyslipidemia, asthma, stroke, emphysema, chronic bronchitis, diabetes, dementia, anxiety, mood disorders, and polypharmacy*	https://doi.org/10.1111/jgs.18957
Kochar 2024	United States	Beers 2019	Disease-specific (PROBE), cut-off < 24	Cross-sectional	Adults ≥ 60 years with inflammatory bowel disease (*n* = 100)*, (median age 68 years interquartile range not reported)*	PIMs OR 1.95 95% CI [1.20;3.19] *Adjusted for age, sex, IBD-type, number of comorbidities and depression*	https://doi.org/10.1007/s10620-023-08250-3

*v* version, *STOPP/START* Screening Tool of Older Persons’ Prescriptions/Screening Tool to Alert to Right Treatment, *PIM* potentially inappropriate medications, *EQ* EuroQol, *SF* Short‐Form Health Survey, *PCS* Physical component summary, *CASP* Control, Autonomy, Self-realisation, and Pleasure tool, *ACOVE* Assessing Quality of Care of Elderly, *MAI* Medication appropriateness index, *FU* follow up, *m* months, *PIP* potentially inappropriate prescribing, *GP* general practitioner, *CIRS-G Score* Cumulative Illness Rating Scale for Geriatrics, *CI* confidence interval, *OR* odds ratio, *RR* relative risk, χ^2^ Chi-square, *DDI* drug-drug interactions, *IBD *inflammatory bowel disease

A cross-sectional study applied the Beers 2019 criteria to identify PIMs and found a significant association between PIMs, and lower physical and mental HRQOL (SF-12) [34]. In contrast, another study reported no statistically significant difference in HRQOL (EQ-5D) among those with PIMs, identified using the Beers 2012 criteria [35]. In the same study, PIMs identified using the Screening Tool of Older Persons' Prescriptions (STOPP) version 1 criteria were significantly associated with poorer HRQOL (EQ-5D) when two or more PIMs were present, with an adjusted regression coefficient of − 0.11 (95% confidence interval (95% CI) [− 0.16; −  0.06]) [35]. These findings suggest that reducing or avoiding PIMs could be a potential strategy for improving HRQOL in older adults.

PPOs have also been linked to HRQOL. In older outpatients, PPOs identified using the Screening Tool to Alert to Right Treatment (START) version 2 criteria were associated with lower HRQOL, as measured by EQ-VAS and EQ-5D, although no such association was found with SF-12 [36]. A decline in HRQOL, as measured by CASP-12, was observed only in those with two or more START criteria [37, 38]. However, in hospitalised older adults, no significant differences in HRQOL (EQ-5D) were found between those with and without PPOs [39].

One study focused specifically on anticholinergic drug burden, rather than overall PIP [40]. It found that higher anticholinergic burden was associated with lower HRQOL (adjusted regression coefficient − 1.84 95% CI [− 3.14; − 0.54]), as measured by CASP-19, even after adjusting for clinical and demographic factors [40].

Specific populations, including those with inflammatory bowel disease [28], diabetes type II [27] and cardiac conditions, such as AF (3.4%) [41] have also been studied. In these populations, PIMs and specifically drug–drug interactions were consistently associated with lower HRQOL, even after adjusting for polypharmacy and comorbidities [27, 28].

These studies predominantly adjust for the number of chronic conditions in their analyses (Table 2). Similar as for polypharmacy, confounding by indication might also be a challenge for assessing the actual association of PIP and HRQOL. Polypharmacy is a known risk factor for PIP, and since polypharmacy and multimorbidity are strongly correlated, this might complicate the assessment of PIP on HRQOL. While adjusting for the number of comorbidities can address some of the underlying health factors, it might not fully capture the complexity of multimorbidity and its influence on HRQOL [20]. A more nuanced approach would be to examine multimorbidity profiles, focusing on clusters of co-occurring diseases. If a significant association is still observed between PIP and HRQOL after accounting for these multimorbidity profiles, it may suggest that the quality of prescribing plays a more substantial role in HRQOL, independent of the underlying health conditions. Further investigation is needed to determine whether incorporating multimorbidity profiles, rather than the number of chronic conditions, offers a more accurate approach. It is also important to be cautious of multicollinearity between polypharmacy, PIP and/or multimorbidity, as it can lead to inaccurate associations.

Overall, only one study has examined the association between the quality of prescribing and HRQOL in older adults with AF, highlighting the need for further research. Moreover, the findings suggest that the quality of prescribing, independent of polypharmacy, may significantly influence HRQOL. This raises the question of whether targeted interventions to improve prescribing quality could lead to better HRQOL.

### Interventions to improve quality of prescribing

Conducting a medication review is necessary to improve prescribing quality. A medication review is defined as “a structured evaluation of a patient's medicines with the aim of optimising medication use and improving health outcomes, which entails detecting drug-related problems and recommending interventions” [42]. Interventions to improve the quality of prescribing differ significantly in design (Table 3).

**Table 3:** Interventions to improve quality of prescribing and its effect on health-related quality of life (HRQOL)

Author, year	Country	Tool to assess quality of prescribing	Tool to assess HRQOL	Type of intervention	Study design, FU	Study population	Effect of intervention on HRQOL	DOI
Hanlon 1996	United States	MAI	SF-36	Clinical pharmacist-driven	RCT, 12 m	Outpatients ≥ 65 years with polypharmacy Intervention (*n* = 105) and control (*n* = 103)	No statistical difference in HRQOL was observed. Proportions for individual dimensions were reported at baseline and after the intervention, but no statistical tests were conducted	https://doi.org/10.1016/S0002-9343(97)89519
Bladh 2011	Sweden	Computer support system (MiniQ)	Self-rated global health, EQ-5D-3L, EQ-VAS	Clinical pharmacist-driven *with medication review performed at multiple time points*	RCT, 6 m	Hospitalised patients intervention (*n* = 95) and control (*n* = 109), (82 [75–82] years)	Per-protocol analysis revealed significantly better HRQOL in the intervention group as measured by global health (mean ± SD (3.14 ± 0.87) vs (2.77 ± 0.94), *p* = 0.02) but not with the EQ-5D utility score	https://doi.org/10.1136/bmjqs.2009.039693
Twigg 2015	England	STOPP/START	EQ-5D-5L	Community pharmacist-driven	Prospective, pre-post interventional study, 6 m	Community older adults ≥ 65 years with polypharmacy (≥ 4) (*n* = 620)	A mean change in EQ-5D score of 0.025 95% CI [0.007; 0.042) was reported No results were reported for individual dimensions	http://doi.org/10.1111/ijpp.12196
Van der Linden 2017	Belgium	RASP list	EQ-5D-3L	Pharmacist-driven	Monocentric, prospective controlled trial, 3 m	Hospitalised patients Intervention (*n* = 117) and control (*n* = 97), *(84.5* ± *4.8 years)*	No significant difference in individual dimensions was found between the groups, but the EQ-5D index increased significantly in the intervention group (0.358 ± 0.016) compared to the control group (0.294 ± 0.018), with a difference of 0.064 points (SE 0.024; *p* = 0.008)	http://doi.org/10.1007/s40266-016-0424-8
Salisbury 2018	England and Scotland	NR	EQ-5D-5L	Pharmacist-driven multidisciplinary 3 dimensional approach	Cluster-randomised trial, 15 m	Primary care adults with ≥ 3 chronic conditions intervention (*n* = 749, 70.7 ± 11.4 years) control (*n* = 797, 71.0 ± 11.6 years)	No significant difference observed between control and intervention. No information on individual dimensions was provided	http://doi.org/10.1016/S0140-6736(18)31308-4
Cardwell 2020	Ireland	Self-developed tool	EQ-5D-5L EQ-VAS	General practice pharmacist-driven	Non-randomised pre-post pilot study, 6 m	Primary care older adults (*n* = 96),* (77.7* ± *6.4 years)*	There was a statistically significant mean difference of -0.056 95%CI [− 0.11;− 0.002] with EQ-5D score, while HRQOL measured by EQ-VAS increased with the same level (0.06, 95% CI [0.02;0.10]) No information on individual dimensions was provided	https://doi.org/10.1136/bmjopen-2019-035087
Almutairi 2020	United Kingdom, Ireland, Finland, Australia	STOPP/START, other studies did not report a specific tool	SF-12, QOL-AD, 15D	Pharmacist-driven Nurse-driven	Systematic review of RCT (*n* = 5 HRQOL studies), 12 m	Older adults in RACF, *(age range of 81.2 to 87.2 years)*	The pooled analysis did not report a statistically significant difference	https://doi.org/10.1186/s12877-020-01634-4
Roughead 2022	Australia	NR	EQ-5D-5L	Pharmacist-driven *medication review—every 8 weeks*	Multicentre RCT, 12 m	Older adults in aged care facilities taking ≥ 4 medicines or ≥ 1 anticholinergic drug Intervention (n = 120) and Control (n = 128)*, (median age 87 years interquartile range not reported)*	No significant difference in HRQOL was observed, and no information on individual dimensions was provided	https://doi.org/10.1093/ageing/afac092
Holland 2023	England, Scotland, Northern Ireland	STOPP/STARTv2 criteria	EQ-5D-5L	Pharmacist independent prescriber-driven *medication review was performed on a weekly basis*	Cluster RCT, 6 m	Care home residents aged ≥ 65 years Intervention (*n* = 449) and Control (*n* = 427)	No significant difference in HRQOL was observed, and no information on individual dimensions was provided	https://doi.org/10.1136/bmj-2022-071883
Yaacob 2024	Turkey, Japan, US, Germany, US, Singapore, Netherlands	NR	EQ-5D, and SF-12	Pharmacist-driven	Scoping review (n = 8 HRQOL studies)	Older adults ≥ 65 years	RCT studies (*n* = 3) reported no significant effect on HRQOL. The remaining studies were quasi-experimental and reported positive influence on HRQOL	https://doi.org/10.4082/kjfm.23.0220
Hassanzadeh 2024	Germany	DSS Medi-check	EQ-5D-5L, EQ-VAS	Pharmacist-driven DSS-supported	Prospective, pre-post interventional study, 6 m	Adults on antithrombotic therapy (*n* = 87)*, (71* ± *14.2 years)*	EQ-VAS improved from 67.3 ± 14.4 to 73 ± 13.4 EQ-5D score improved from 0.81 ± 0.2 to 0.88 ± 0.1	https://doi.org/10.3389/fphar.2024.1194201
Hurley 2024	Ireland	STOPPFrail	EQ-5D-5L and EQ-VAS	Pharmacist-driven *deprescribing interventions*	Unblinded non-randomised study, 6 m	Frail older adults in nursing homes aged ≥ 65 years (*n* = 99)	No significant difference in HRQOL was observed, and no information on individual dimensions was provided	https://doi.org/10.1016/j.jamda.2024.105122
Roncal-Belzunce 2024	Netherlands, Spain, Ireland, Australia, United Kingdom, Germany, Sweden, Taiwan	NR	SF-12/36, EQ-5D	Pharmacist-driven	Systematic review of multidisciplinary interventions RCT (*n* = 12 HRQOL studies), 3–12 m	Community-dwelling older adults with polypharmacy	Pooled prevalence of eight studies resulted in non-significant difference between intervention and control. The other three studies also reported no significant results. Only one study reported significant improved scores in vitality and mental health with the SF-36 tool	https://doi.org/10.1016/j.arr.2024.102317
Okafor 2024	Australia	Structured deprescribing algorithm	EQ-5D-5L, carer-reported	Pharmacist-driven *deprescribing intervention With the help of a structured deprescribing algorithm*	RCTs, 12 m	Frail older people in RACF Blinded intervention (*n* = 102), open intervention (*n* = 101) and Control (*n* = 100),* (85.0* ± *7.5 years)*	No significant difference in HRQOL was observed, and no information on individual dimensions was provided	https://doi.org/10.1016/j.jamda.2023.12.016
Potter 2016	Australia	List based on Beers, Australian list of prescribing indicators and inappropriate medication use, and studies	QOL-AD and EQ-5D	Physician (GP)-driven *deprescribing intervention*	Open RCT, 12 m	Older adult ≥ 65 years in RACF intervention (*n* = 47) and control (*n* = 48)	No significant difference in HRQOL was observed with either tool, and no results were reported for individual dimensions	http://doi.org/10.1371/journal.pone.0149984
Anderson 2020	Australia	Self-developed tool	EQ-5D-5L—telephone version EQ-VAS	Physician (GP)-driven *multifaceted intervention*	Controlled pre-post design, 18 weeks	Community dwelling older adults ≥ 65 years intervention (*n* = 78) and control (*n* = 67)	No significant differences were found in any of the EQ-5D-5L domains	https://doi.org/10.1111/jgs.16273
O'Mahony 2020	Iceland, Ireland, Scotland, Spain, Belgium, Italy	Software based on STOPP/STARTv2, DDI, and information from local databases	EQ-5D-3L	Physician-driven *SENATOR software-guided medication review. Primary researchers provided the clinicians with the SENATOR software-generated recommendations*	Randomised controlled clinical trial, 3 m	Hospitalised older adults ≥ 65 years with multimorbidity and polypharmacy intervention (*n* = 772) and control (*n* = 765)	No significant difference in HRQOL was observed between intervention and control, and no information on individual dimensions was provided	http://doi.org/10.1093/ageing/afaa072
Mccarthy 2022	Ireland	STOPP/STARTv2	EQ-5D-5L	Physician (GP)-driven	Cluster randomised controlled trial, 6 m	Primary care older adults ≥ 65 years with multimorbidity and excessive polypharmacy (≥ 15 medications) intervention (*n* = 208) and control (*n* = 196)	No significant difference in HRQOL was observed after adjusting	https://doi.org/10.1371/journal.pmed.1003862
Saeed 2022	United States, Belgium, Ireland	STOPPFrail	ICECAP-O, QUALIDEM	Physician-driven *pharmaceutical care supplemented by STOPPFrail deprescribing plan*	Systematic review of (non)- RCT (*n* = 1 HRQOL study), 3 m	Frail older patients in adults aged ≥ 65 years in secondary or acute care settings	No significant difference in HRQOL was observed, and no information on individual dimensions was provided	https://doi.org/10.1007/s11096-021-01354-8
Jungo 2023	Switzerland	Electronic clinical decision support system (STRIPA) based on STOPP/STARTv2	EQ-5D-5L, EQ-VAS	Physician (GP)-driven *medication review with STRIPA, an electronic clinical decision support system*	Cluster randomised clinical trial, 12 m	Older adults ≥ 65 years with ≥ 3 chronic conditions and ≥ 5 chronic medications intervention (*n* = 160) and control (*n* = 163)	No significant difference in HRQOL was observed, and no information on individual dimensions was provided	http://doi.org/10.1136/bmj-2022-074054
Olesen 2024	Denmark	NR	Self-reported health on a scale from 1 to 10	Physician (GP)-driven *deprescribing intervention incorporated in a chronic care model*	Pre-post study, 3-4 m	Care home residents and community-dwelling adults (*n* = 105), (81 [71–88] years)	Mean self-reported health status increased (0.55 95% CI [0.22; 0.87])	https://doi.org/10.1111/bcpt.13925
Cole 2023	Europe, Canada, United States, Hong Kong, Malaysia, Australia	STOPPv1, STOPPv2, Beers 1997, Beers 2012, PRISCUS	EQ-5D, SF-36, SF-12, 15D, QUALIDEM, ICECAP-O	Multidisciplinary	Systematic review of randomised trials (*n* = 16 HRQOL studies), 3-12 m	Older adults ≥ 65 years	5 out of 16 studies reported an improvement in HRQOL. Author identified a low certainty of evidence	https://doi.org/10.1002/14651858.cd008165.pub5
Braithwaite 2023	Netherlands, United Kingdom, Finland, Norway, Australia	STOPP/START For the other studies a tool was not reported	EQ-5D and SF-36	Multidisciplinary *multistep medication review intervention*	Systematic review of RCT (*n* = 3)	Older adults ≥ 65 years prescribed anticholinergic medications	Pooled results from two studies using EQ-5D showed a mean difference of 0.04 95% CI [− 0.04;0.12], with no information on individual dimensions	https://doi.org/10.1093/ageing/afad176
Zhou 2023	Europe, Canada, United States, Singapore, Malaysia, Australia	NR	SF-12/36, 15D, EQ-5D-3L, EQ-VAS	Multidisciplinary *deprescribing interventions, educational intervention, comprehensive geriatric assessment*	Systematic review of RCT (*n* = 10 HRQOL studies)	Older adults ≥ 60 years	One study reported an improvement in vitality (SF-36) and Pitkälä, 2014 reported a slower decrease in HRQOL in the intervention group. Other studies reported no significant difference	https://doi.org/10.1016/j.jamda.2023.07.016
Bloomfield 2020	Spain, Netherlands, Germany, United Kingdom, Finland, Denmark, Sweden, Malaysia, United States	NR	EQ-5D SF-12/36	Multidisciplinary *deprescribing interventions, comprehensive medication review, educational interventions, DSS*	Systematic review of (clustered) (R)CT, 3-6 m	Community-dwelling older adults ≥ 65 years	No differences were reported between groups in the majority of studies, and the author noted a low certainty of evidence	http://doi.org/10.1007/s11606-020-06089-2
Pitkälä 2014	Finland	Beers 2003, Anticholinergic risk scale, Swedish list of anticholinergic medications	15D	Multidisciplinary *an educational intervention was implemented to train nursing staff on conducting medication reviews*	RCT, 12 m	Older adults in RACF intervention (*n* = 118, 82.9 ± 7.5 years)) and control (*n* = 109, 83.5 ± 6.9 years))	HRQOL declined more slowly in the intervention group (− 0.038 95% CI [− 0.054;− 0.022]) than in the control group (− 0.072 95% CI [− 0.089;− 0.055]) *Adjusted for age, sex, and comorbidities* The dimensions Breathing, Sleeping and Speech showed significant differences in favor of the intervention group	http://doi.org/10.1016/j.jamda.2014.04.002
Pruskowski 2019	Australia, Norway, Belgium, United States, Spain, Netherlands, Japan, Sweden	NR	SF-12/36, EQ-5D-3L, QOL-AD, McGill QOL	Population-specific *deprescribing interventions*	Systematic review (*n* = 10 assessing HRQOL)	Older adults ≥ 65 years and those with life-limiting conditions	Only 2 out of 10 studies found a statistically significant association with HRQOL: in older adults with serious illness a statistically significantly higher total HRQOL was reported in the statin discontinuation group (*n* = 381; McGill QOL score 7.11 vs. 6.85; *p* = 0.04) A non-randomised, pseudo-experimental study of long-term benzodiazepine users found improved HRQOL at 24 m in those who discontinued and maintained abstinence (*n* = 51; mean SF-12 score 49.27 vs. 40.72; *p* = 0.02)	https://doi.org/100.1007/s40266-019-00717-1
Ritchie 2024	United kingdom	Atrial Fibrillation Better Care (ABC) pathway	EQ-5D-5L, AFEQT	Population-specific pharmacist-driven	Individually randomised prospective pilot and feasibility study, 6 m (*n* = 21)	Older adults ≥ 65 years with atrial fibrillation in care homes intervention (*n* = 11) and control (*n* = 10)	No conclusions could be drawn because (i) the study was underpowered and (ii) most residents' medications were already been optimised	https://doi.org/10.1186/s12877-023-04527-4

*v* version, *STOPP/START* Screening Tool of Older Persons’ Prescriptions/Screening Tool to Alert to Right Treatment, *EQ* EuroQol, *SF* Short‐Form Health Survey, *PCS* Physical component summary, *CASP* Control, Autonomy, Self-realisation, and Pleasure tool, *ACOVE* Assessing Quality of Care of Elderly, *MAI* Medication appropriateness index, *FU* follow up, *DDI* drug-drug interactions, *RASP* Rationalization of Home Medication by an Adjusted STOPP in Older Patients, *NR* not reported, *DSS* Decision support system, *QOL-AD* Quality of Life in Alzheimer’s Dementia, *RCT* randomised controlled trial, *m* month, *RACF* residential aged care facilities, *GP* general practitioner, *SD* standard deviation, *CI* confidence interval, *AFEQT* Atrial Fibrillation Effect on Quality-of-Life. The average age is mentioned in the “Study population” column for studies that did not exclusively include adults aged 65 years or older

### Pharmacist-driven interventions

**Fig. 1:**
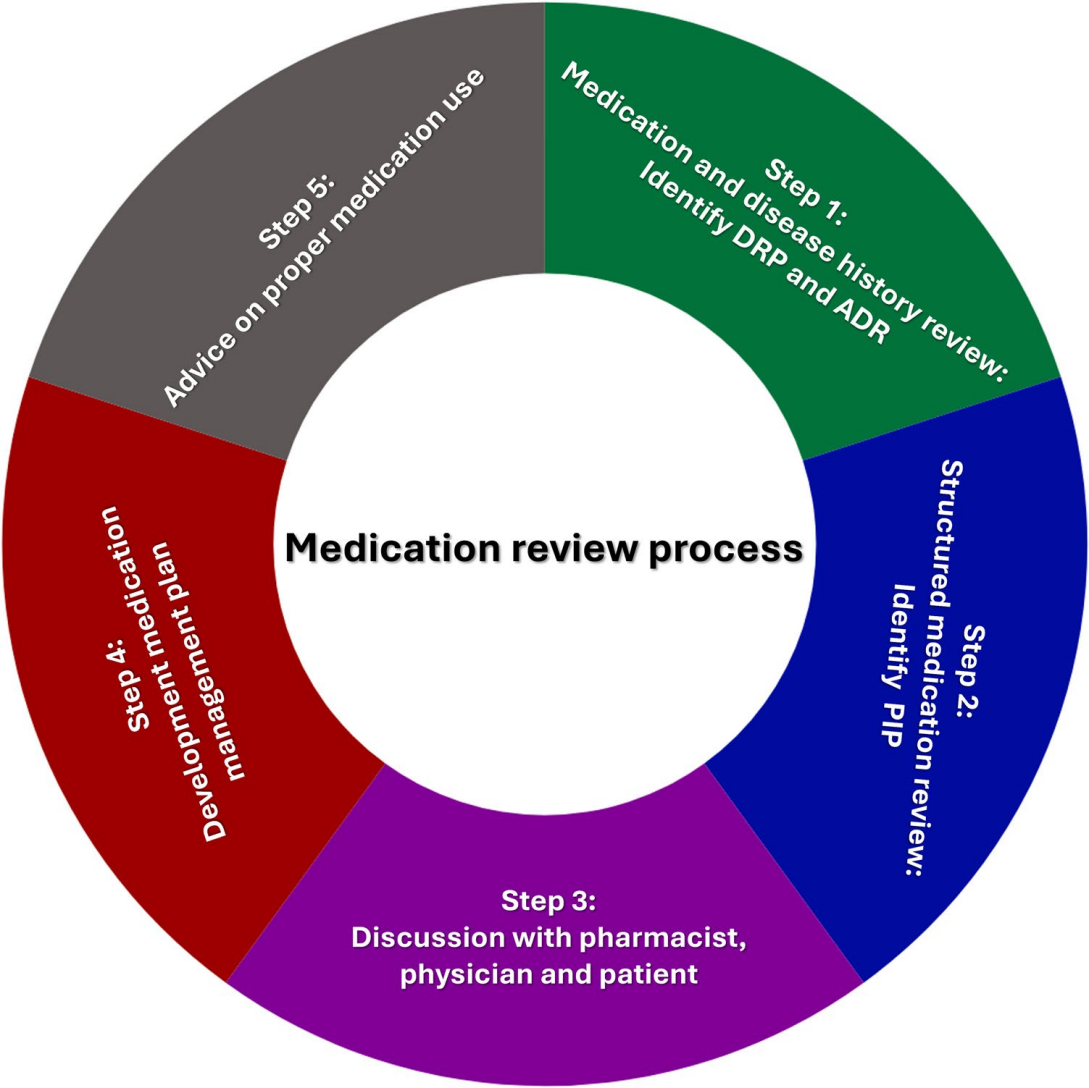
Methodological approach to a medication review process. *DRP* Drug related problems, *ADR* adverse drug reactions, *PIP* potentially inappropriate prescribing. Source: adapted from Petrovic et al. (2016) [43]

Pharmacist-driven medication reviews have gained attention. Typically, the medication review process begins with the pharmacist reviewing the patient’s medication history to identify current and past medications, including any previous adverse drug reactions (ADRs) and drug-related problems (DRPs) (Fig. 1). Following this initial assessment, a structured medication review is conducted to evaluate the medication regimen for potential misuse, underuse, or overuse. Any identified DRPs are discussed with both the physician and the patient, when possible. Finally, a medication management plan is developed, and the pharmacist advises the patient on proper medication use [43].

A recent scoping review indicated that 90% of the included studies showed an overall reduction in DRPs and PIMs [44]. While the three RCTs found no statistically significant difference, the five quasi-experimental and observational studies, reported a significant positive impact on HRQOL [44]. A monocentric prospective controlled trial reported a significant improvement in HRQOL (EQ-5D-3L) compared to baseline in geriatric inpatients following a pharmacist-driven intervention [45].

A systematic review identified only one study assessing the effectiveness of a clinical pharmacist-driven medication review, supported by a computer system, on HRQOL in hospitalised patients [42, 46]. This intervention involved collaboration between clinical pharmacists, geriatricians, patients, and their general practitioner (GP). While HRQOL, measured by EQ-5D and EQ-VAS, showed no statistically significant improvement compared to the control group—likely due to a high dropout rate—the intervention group reported a statistically significant increase in self-rated global health scores [46]. A primary care cluster-randomised trial evaluating a patient-centred care model for older adults with multimorbidity found no statistically significant difference in EQ-5D-5L scores. Notably, only 49% of the intervention participants (*n* = 749) received the intended two comprehensive multidisciplinary reviews. In addition, while a full pharmacist-driven medication review was conducted, the number of medications was used as a measure of PIP rather than focusing on the identified PIMs and PPOs. These factors might have contributed to the lack of significant findings [47].

Similarly, another study found that a pharmacist-driven medication review, supported by a clinical decision support system, improved HRQOL, as measured by EQ-5D-5L and EQ-VAS, in polymedicated community-dwelling older adults. This non-randomised, unblinded study emphasised patient education and involvement as central and successful components of the intervention [48]. A community pharmacy multi-consultation intervention also showed positive trends in HRQOL in polymedicated older adults with a mean change in EQ-5D score of 0.025 95% CI [0.007; 0.042]. This intervention provided holistic care by also addressing fall risk, pain management, medication adherence, information on public health interventions and referrals to the patient’s GP for necessary medication changes [49].

The low application rate of pharmacist recommendations by the GPs in frail older nursing home residents (*n* = 99), potentially led to no significant HRQOL differences [50]. Interestingly, a general practice-based pharmacist intervention reported a decrease in EQ-5D, but an increase in overall health perception (EQ-VAS). The decrease in EQ-5D was attributed to the use of an external value set, derived from the general Irish population, which may not fully reflect the specific health preferences of the study participants. This could explain the discrepancy between the lower EQ-5D index score, and the higher EQ-VAS scores reported. Although the proportions for the dimensions were reported, only the difference in overall EQ-5D value was assessed [51].

Pharmacist-driven medication reviews, particularly those involving patient education and multidisciplinary collaboration, have demonstrated potential in reducing PIP and DRPs and improving HRQOL, though results vary depending on the study design and the tools used to assess HRQOL. Further research is needed to optimise these interventions and evaluate their long-term effects on HRQOL.

### Physician-driven interventions

Physician-driven judicious deprescribing interventions often showed limited impact on HRQOL. One study found no significant improvement in HRQOL (EQ-5D-5L) after an 18-week follow-up in an exploratory controlled pre-post-trial with polymedicated community-dwelling older adults [52]. Similarly, the SPPiRE cluster randomised controlled trial assessed a physician-driven medication review in older polymedicated adults (≥ 11 medications) with multimorbidity [53]. The intervention demonstrated no statistically significant difference in EQ-5D-5L scores at 6 months, compared to usual care. However, within the intervention group, a statistically significant reduction in the number of prescribed drugs was observed [53].

In line with these findings, another study reported no significant differences in HRQOL (EQ-5D-5L) after 12 months, in an intervention where general practitioners used STRIPA, a clinical decision support system, based on the STOPP/START version 2 criteria. The low implementation rates of the recommendations (one per patient) might have contributed to the lack of observed effect [54]. The SENATOR trial, which also applied the STOPP/START version 2 criteria in a software-assisted medication review, found no statistically significant difference in HRQOL between intervention and control groups in hospitalised polymedicated older adults with multimorbidity, possibly due to the very low implementation rate (15%) of the recommendations by clinicians. Notably, in this study, the primary researchers provided software-generated recommendations to the treating physicians [55]. In contrast, a deprescribing intervention performed within a chronic care model did improve self-reported overall health perception, using a scale from (1–10), compared to baseline [56].

Overall, physician-driven deprescribing interventions generally show limited impact on HRQOL, possibly due to low implementation rates. However, interventions within chronic care models and a multidisciplinary approach may improve HRQOL.

### Multidisciplinary interventions in older adults with polypharmacy

Two studies reviewed RCTs that assessed the effectiveness of multidisciplinary interventions targeting potentially inappropriate prescribing, in community-dwelling older adults with polypharmacy. The reviews reported improvements in medication appropriateness, adherence, and reduction in medication numbers and associated costs. However, these studies demonstrated only slight to no improvements in HRQOL, as measured by generic tools, and the pooled analysis resulted in no significant association. The authors also emphasised the low certainty and high risk of bias in the included studies [57, 58]. Similarly, in a different systematic review, studies using the EQ-5D and SF-12/36 scales found no significant difference in HRQOL between groups [59]. In contrast, three studies using the 15D scale showed mixed results: with two multidisciplinary approaches reducing decline in HRQOL [59]. Another systematic review found similar results in 11 studies, with no differences reported between groups in most cases and a low certainty of evidence [60].

Multidisciplinary interventions improve medication management but show limited impact on HRQOL. The modest improvements in HRQOL, coupled with the low certainty and high risk of bias in existing studies, highlight the need for further research and more robust intervention designs.

### Interventions in residential aged care facilities

Interventions in aged care facilities often showed no significant HRQOL improvement. A systematic review on medication optimisation in residential aged care facilities reported improvements in medication appropriateness but no significant differences in health-related outcomes [61]. Although five studies assessed HRQOL using generic tools (SF-12 and 15D) and disease specific tools (Quality of life in Alzheimer’s Dementia (QoL-AD)), the pooled analysis showed no significant differences in HRQOL [61]. However, one study using a multidisciplinary approach to deprescribe potentially harmful medications, demonstrated a slower decline in HRQOL, as assessed by 15D [61, 62].

In a multicentre RCT (ReMinDARtrial) involving polymedicated non-frail older adults in aged-care facilities, no significant changes in HRQOL or incident adverse events were observed, likely due to the small sample size [63]. Similar results were reported in an intensive cluster RCT, despite a significant reduction in the Drug Burden Index [64]. Regardless of multidisciplinary team efforts to deprescribe medications, 41% of identified medications in frail older adults residing in care facilities were either not attempted or failed to be withdrawn [65]. Similarly, a comprehensive multidisciplinary deprescribing intervention, using an algorithm, did not impact HRQOL after 12 months, likely due to the small sample size and, therefore, being underpowered [66]. Improving quality of prescribing also failed to alter HRQOL in frail older adults nearing end-of-life [67].

Interventions in aged care facilities demonstrated limited impact on HRQOL, with some studies noting slower declines or no significant differences. Challenges in implementing deprescribing efforts and small sample sizes may contribute to these findings. Future research should aim for larger studies to better evaluate intervention effectiveness.

### Interventions aimed at specific drug classes and health conditions

Several studies also targeted specific drug classes or populations. A pooled analysis of three RCTs on anticholinergic drug burden, showed no statistically significant difference in HRQOL [68]. However, one study included in the review reported improvements in HRQOL among nursing home residents following an intervention by geriatricians and pharmacists [68]. In a systematic review of deprescribing interventions, including (non)-RCTs, only two out of ten studies reported a significant improvement in HRQOL [69]. These specific studies focused on discontinuing statins in those with short life expectancy, and benzodiazepines for over 24 months. Deprescribing interventions did not improve HRQOL in studies specifically targeting older adults with dementia and those with malignant tumours [69].

Only one study focused on older adults with AF. It consisted of a pharmacist-led randomised prospective pilot and feasibility study in older care home residents with AF. Based on an AF integrated care pathway (ABC), the pharmacist developed medication recommendations, which were communicated to the general practitioner. Although HRQOL was assessed, using both generic (EQ-5D) and disease-specific (AFEQT) assessment tools, the study was underpowered, and most residents’ medications were already optimised, preventing any conclusions from being drawn [70].

Overall, the results were inconsistent, with most studies showing no statistically significant improvement in HRQOL (Table 3). Additionally, no information was provided on the dimensions of the EQ-5D assessment tool; only the overall score was reported and compared. The absence of a difference in the overall score does not necessarily imply that the intervention had no impact on the EQ-5D dimensions.

Given the high prevalence of PIP in older adults with AF and the limited evidence available, further research is essential to better understand the implications for their HRQOL and to identify interventions that can improve both prescribing practices and patient outcomes. Barriers to effective interventions and limitations of study designs have been identified, which could be addressed in future research to ensure the development of more robust, high-quality interventions.

### Barriers and facilitators of effective prescribing interventions

**Fig. 2:**
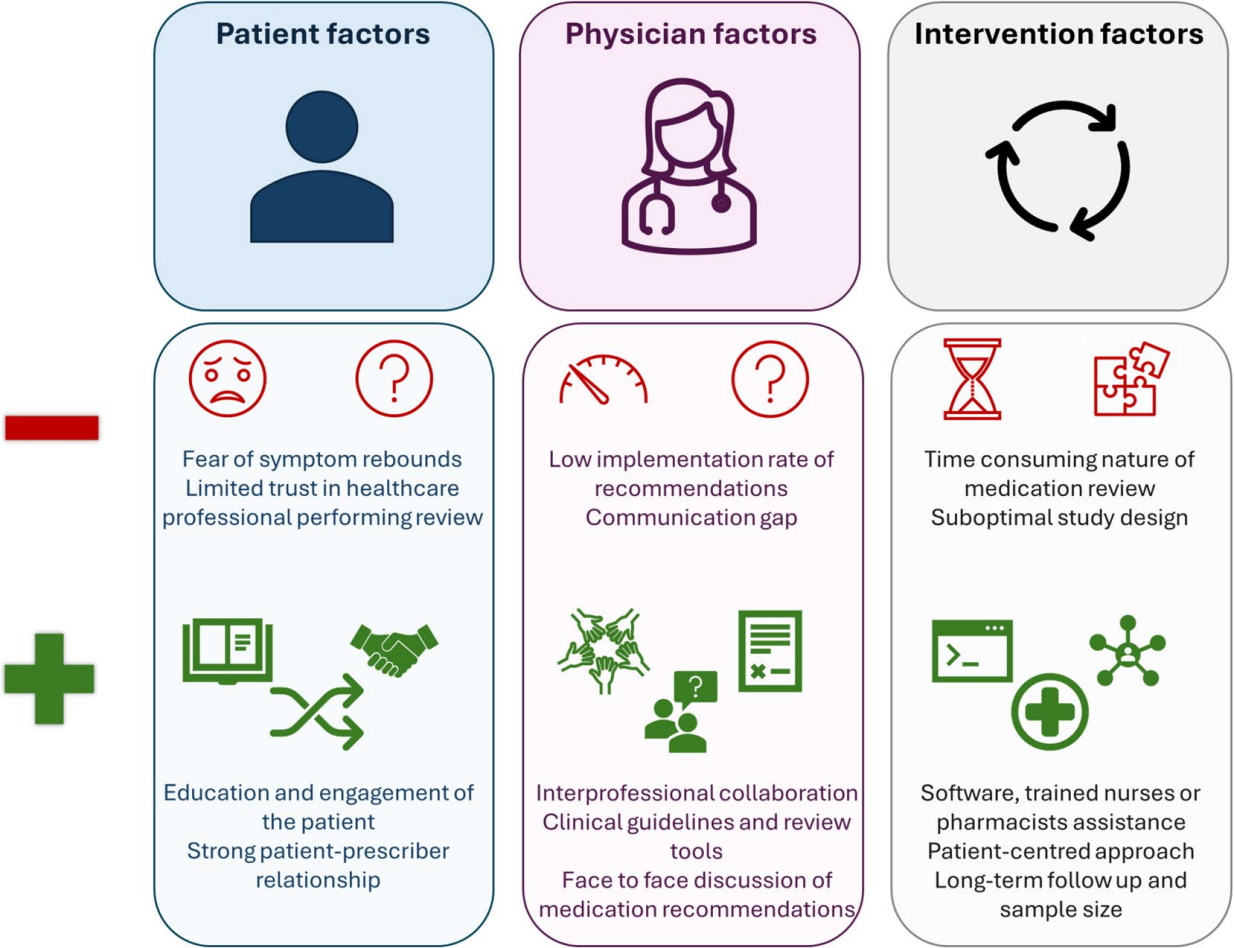
Barriers and facilitators of prescribing interventions

Barriers encountered in intervention studies were patient-, prescribing physician- and intervention- related (Fig. 2).

### Patient-related factors

Patient refusal, often due to fear of symptom rebound was a barrier to deprescribing [42, 65, 71]. However, the reasons for refusal were not always fully explored. Moreover, patients’ relationship with their medications and the involvement of their caretakers also play an essential role [72]. One study found that patients showed apprehension when pharmacists conducted medication reviews [48], while another emphasised that the patient–prescriber relationship was valued more than the prescriber's title [73]. By ensuring relational continuity, patients build trust in their healthcare professionals, who can then tailor recommendations based on the patient’s specific needs and best interests. Additionally, monitoring the medication changes might alleviate patient fears by demonstrating that potential harms will be managed. This approach also provides time for patients to adjust to prescribing changes. Educating and actively engaging patients and/or their caregivers in shared decision-making can help overcome their reluctance, thereby facilitating the process [42, 48]. Targeting and tailoring interventions to specific patients, such as those with polypharmacy, high treatment burden, specific diagnoses or multimorbidity profile might improve efficacy [74, 75].

### Prescribing physician-related factors

The implementation rate of medication changes to improve the quality of prescribing ranges from 15% to 58.5% [54, 55, 65, 70, 76]. Medications with low withdrawal success include benzodiazepines, antidepressants, analgesics, laxatives, proton pump inhibitors, non-steroidal anti-inflammatory drugs, and beta-blockers [65, 71, 77]. In contrast, one study reported successful withdrawal of these medications, except for antidepressants [74]. Physicians do not follow deprescribing recommendations for a plethora of reasons, including perceived benefit of current prescriptions, it being unsuitable for the patient, presence of an indication, medications being prescribed by specialists, symptom recurrence and/or negative past experiences. Moreover, physicians express that they would have more trust in a pharmacist’s medication review if the pharmacist had regular communication with the patient [78]. These findings indicate gaps in patient involvement, interprofessional collaboration and communication [46, 54, 71, 74, 78].

Guidelines and review tools can encourage deprescribing, but their application is challenging in older adults with multimorbidity, as it is time-consuming, requires experience and lacks adequate incentives or administrative support [53, 72, 78, 79]. Physicians’ decisions were also dependent on patients’ clinical profile [80]. For instance, in a case-vignette study, physicians were more likely to deprescribe CV preventive medications (such as statins and aspirin) for non-CV patients compared to CV patients [80]. Additionally, face-to-face discussions might be more effective for implementing medication recommendations compared to remote communication [50]. The level of experience and knowledge of physicians plays a significant role in their comfort level with deprescribing, particularly when guidelines are not available. Managing medications in older adults requires specialised skills and knowledge. Multidisciplinary teams can help address these challenges, reassuring physicians and ensuring informational continuity, which is also a barrier to deprescribing medications prescribed by other physicians [72].

### Intervention-related factors

A systematic review on deprescribing interventions reported improvements in HRQOL in polymedicated older adults when HRQOL was the primary outcome. However, no improvements were observed when HRQOL was considered a secondary outcome [77]. The intervention studies included in the review adjusted mainly for age, sex, multimorbidity, and polypharmacy, while other factors—such as living arrangements, access to healthcare, socioeconomic status, and environmental factors like the quality of care in nursing homes or hospitals—may also influence the impact of interventions on HRQOL.

Although the generic EQ-5D scales were commonly used, their sensitivity to detect changes in HRQOL following deprescribing remains uncertain [81]. HRQOL tools may not be interchangeable due to differences in their theoretical design and composition. A study involving patients with stable coronary heart disease compared EQ-5D and SF-12 converted to SF-6D utility scores [82]. While a mild correlation was observed, significant differences in outcomes between these instruments were noted, indicating they are not interchangeable [82]. Furthermore, evaluating changes in the individual dimensions of HRQOL, rather than focusing solely on composite outcomes, could provide deeper insights into specific areas where improvements or declines occur, allowing for the identification of factors most relevant to patients [81].

Since over-the-counter (OTC) medications are also linked to lower HRQOL and adverse drug events [83, 84], it is crucial to review both prescribed and OTC medications. Polymedicated patients and their caregivers prioritise medications that alleviate symptoms like pain, anxiety, and depression, while adherence is often tied to perceived improvements in clinical values, such as blood pressure and cholesterol levels. A major reason for non-adherence includes side-effects impairing their HRQOL (dizziness, fatigue, cognitive impairment, and gastrointestinal upset) [73]. Patients are often informed about the reasons for taking their medications, but not about potential side effects [78, 85]. This omission may result from a fear of alarming patients or from time constraints during consultations, which restrict the information conveyed and processed [85]. The time-consuming nature of comprehensive medication reviews further complicates this issue, as healthcare professionals are often forced to prioritise tasks within the limited consultation time available. This affects their cognitive and emotional capacity to weigh the potential benefits and harms of medication changes, especially in the absence of appropriate guidelines [72].

Incorporating specialty-trained professionals, such as clinical pharmacists specialised in older adult care, can help reduce this burden [42, 46, 50]. Clearly defining the roles and responsibilities for conducting medication reviews and managing the deprescribing process can help foster ownership and increase engagement in the process [72]. While software tools can expedite the process, they should support, not replace, interprofessional collaboration among pharmacists, specialists, GPs, and nurses. Long-term follow-up and a patient-centred approach is essential for sustained medication changes [42, 49, 54, 77, 86, 87].

### Future directions and conclusions

Studies have reported lower HRQOL in older adults with AF compared to those without, attributing this disparity to high symptom burden and multimorbidity [20, 88–92]. While several studies have examined HRQOL in relation to rhythm versus rate control or anticoagulant use [93–95], the relationship between HRQOL and appropriate prescribing in AF patients remains underexplored, despite the high prevalence of PIP (73.2%) [96]. Future research should carefully select the HRQOL assessment tool, as generic measures like EQ-5D and SF-12 may not fully capture the specific impact of AF-related symptoms or comorbidities [97]. Disease- or medication-specific scales might provide more sensitive indicators of HRQOL changes [81, 98–100]. However, medication-specific scales have yet to be implemented in intervention studies [81]. Given that older adults with AF are characterised by multimorbidity, incorporating both generic and disease-specific instruments could provide a more comprehensive assessment of the impact of disease and treatment on HRQOL, as well as on the cost-effectiveness of prescribing interventions [3].

Adopting a multidisciplinary, integrated, patient-centred intervention is essential for sustainable and appropriate prescribing practices and for potentially improving HRQOL. AF patients, caregivers and healthcare practitioners reported patient education, effective communication, medication reviews and quality of life as key elements for their care management [101]. Appropriate pharmacotherapy should be combined with a comprehensive assessment of patients’ clinical and functional parameters, considering their HRQOL and actively involving them in both the management of their pharmacotherapy and treatment decisions. Patient-centred care, supported by the integration of expertise from various healthcare professionals is essential to address the medical complexity of older adults, including those with AF.

## Supplementary Information

Below is the link to the electronic supplementary material.

Supplementary file1 (DOCX 16 KB)

Supplementary file2 (DOCX 18 KB)

Supplementary file3 (DOCX 36 KB)

## Data Availability

All data generated or analysed during this study are included in this published article.
