# Supplementary material for: Quality of prescribing and health-related quality of life in older adults: a narrative review with a special focus on patients with atrial fibrillation and multimorbidity
Source: Eur Geriatr Med. 2025 Mar 9;16(4):1137–61. doi: 10.1007/s41999-025-01175-2 (PMC12378476; doi:10.1007/s41999-025-01175-2)
Supplement: Supplementary file 1 — Supplementary file1 (DOCX 16 KB) [file 41999_2025_1175_MOESM1_ESM.docx]

**Quality of prescribing and Health-Related Quality of Life in Older Adults: A Narrative Review with a Special Focus on Atrial Fibrillation and Multimorbidity**

European Geriatric Medicine

*Cheima Amrouch^1,2^, Deirdre A. Lane^3,4,5^, Amaia Calderón-Larrañaga^6,7^, Mirko Petrovic^1*^, Delphine De Smedt^2*^, on behalf of the AFFIRMO investigators*

*1 Department of Internal Medicine and Paediatrics, Ghent University, Ghent, Belgium
2 Department of Public Health and Primary Care, Ghent University, Ghent, Belgium
3 Liverpool Centre for Cardiovascular Science, University of Liverpool, Liverpool John Moores University and Liverpool Heart & Chest Hospital, William Henry Duncan Building, L7 8TX, Liverpool, UK
4 Department of Cardiovascular and Metabolic Medicine, Institute of Life Course and Medical Sciences, University of Liverpool, Liverpool, UK
5 Department of Clinical Medicine, Aalborg University, Aalborg, Denmark
6* *Department of Neurobiology, Aging Research Center, Care Sciences and Society, Karolinska Institutet and Stockholm University, Stockholm, Sweden
7* *Stockholm Gerontology Research Center, Stockholm, Sweden
*Shared last author*

[*cheima.amrouch@ugent.be*](mailto:cheima.amrouch@ugent.be)

**AFFIRMO consortium members:**

Søren Påske Johnsen, Gregory Y.H. Lip, Berit Hvidberg Christensen (Aalborg Universitet), Gregory Y.H. Lip, Riccardo Proietti, Deirdre Lane, Martin O’Flaherty, Carrol Gamble, Iain Buchan, Christodoulos Kypridemos, Brendan Collins, Donato Leo (The University of Liverpool), Mirko Petrovic, Delphine De Smedt, Cheima Amrouch (Universiteit Gent), Davide Liborio Vetrano, Amaia Calderón-Larrañaga, Lu Dai (Karolinska Institutet), Stefania Maggi, Marianna Noale (Consiglio Nazionale delle Ricerche), Gheorghe-Andrei DAN, Anca Rodica Dan, Elisabeta Badila, Adrian Catalin Buzea, Raluca Popescu Universitatea de Medicina si Farmacie “Carol Davila” din Bucuresti), Nicola Ferri, Alessandra Buja, Giuseppe Sergi, Vincenzo Stefano Rebba, Caterina Trevisan (Università degli Studi di Padova), Tatjana Potpara (Faculty of Medicine, University of Belgrade), Laura Vivani, Silvia Ananstasia (Moverim Consulting sprl), Alessandro Ferri, Gehad Shehata, Nadia Rosso, Marco Cicerone (Advice Pharma Group srl), Jacek Marczyk (Ontonix), Trudie Lobban (Arrhythmia Alliance), Georg Ruppe (European Union Geriatric Medicine Society aisbl), Benedetta Marcozzi, Federica Censi, Robero Da Cas, Cecilia Damiano (Istituto Superiore di Sanità), Guendalina Graffigna, Caterina Bosio, Lorenzo Palamenghi, Serena Barello (Università Cattolica del Sacro Cuore), Marco Proietti (University of Milan), Aldo Pietro Maggioni, Andrea Lorimer, Donata Lucci (Heart Care Foundation Onlus), Dipak Kalra, Nathan Lea (The European Institute for Innovation through Health Data), John Ainsworth, Charlotte Stockton-Powdrell, Alam Sanaullah (The University of Manchester), Francisco Marín Ortuño, José Miguel Rivera-Caravaca University of Manchester), Francisco Marín Ortuño, José Miguel Rivera-Caravaca, Vanessa Roldán, María Asunción Esteve-Pastor, Cecilia López-García, Pablo Gil-Pérez (Universidad De Murcia), Mariya Tokmakova (Medical University Plovdiv).
