# Supplementary material for: Quality of prescribing and health-related quality of life in older adults: a narrative review with a special focus on patients with atrial fibrillation and multimorbidity
Source: Eur Geriatr Med. 2025 Mar 9;16(4):1137–61. doi: 10.1007/s41999-025-01175-2 (PMC12378476; doi:10.1007/s41999-025-01175-2)
Supplement: Supplementary file 2 — Supplementary file2 (DOCX 18 KB) [file 41999_2025_1175_MOESM2_ESM.docx]

**Quality of Prescribing and Health-Related Quality of Life in Older Adults: A Narrative Review with a Special Focus on Patients with Atrial Fibrillation and Multimorbidity**

European Geriatric Medicine

*Cheima Amrouch^1,2^, Deirdre A. Lane^3,4,5^, Amaia Calderón-Larrañaga^6,7^, Mirko Petrovic^1*^, Delphine De Smedt^2*^, on behalf of the AFFIRMO investigators^§^*

*1 Department of Internal Medicine and Paediatrics, Ghent University, Ghent, Belgium
2 Department of Public Health and Primary Care, Ghent University, Ghent, Belgium
3 Liverpool Centre for Cardiovascular Science, University of Liverpool, Liverpool John Moores University and Liverpool Heart & Chest Hospital, William Henry Duncan Building, L7 8TX, Liverpool, UK
4 Department of Cardiovascular and Metabolic Medicine, Institute of Life Course and Medical Sciences, University of Liverpool, Liverpool, UK
5 Department of Clinical Medicine, Aalborg University, Aalborg, Denmark
6* *Department of Neurobiology, Aging Research Center, Care Sciences and Society, Karolinska Institutet and Stockholm University, Stockholm, Sweden
7* *Stockholm Gerontology Research Center, Stockholm, Sweden
*Shared last author
^§^List of coauthors in Appendix 1*

[*cheima.amrouch@ugent.be*](mailto:cheima.amrouch@ugent.be)

**Concepts incorporated in the search strategy**

| **Concept** | **Search Terms** |
| --- | --- |
| **Concept 1: potentially inappropriate prescribing** | Inappropriate prescri*, overdos*, over-dos*, underdos*, under-dos*, mis-dos*, misdos*, overprescri*, underprescri*, misprescri*, over-prescri*, under-prescri*, mis-prescri*, inappropriate medic*, medication error, medication errors, medications error, medications errors, medicine errors, inappropriate drug, inappropriate drugs, suboptimal prescri*, suboptimal therapy, suboptimal therapies, inadequate medic*, inadequate prescri*, inadequate dosing, inadequate therapy, appropriate prescri*, prescription pattern, prescription patterns, prescriptions patterns, prescriptions pattern, prescribing pattern, prescribing patterns, high-risk medic*, inappropriate use, medication appropriateness, drug appropriateness, pharmacological inappropriateness, drug therapy problem, drug therapy problems, inappropriate/inadequate/suboptimal drug therapy, harmful medic*, prescription appropriateness, prescribing appropriateness, prescription error, prescriptions errors, prescription errors, prescribing error, prescribing errors, medication safety, medications safety, drug safety, drugs safety, medicine safety, medicines safety, safe prescri*, prescri* drug misuse / overuse / underuse , prescri* medic* misuse/overuse/underuse, drug overdose, Potentially Inappropriate Medication list, deprescri*, unnecessary prescri*, potential prescri* omissions, irrational use of medic*, irrational use of drugs, prescribing cascade, prescription cascade, inappropriate polypharmacy, abberant prescri* |
| **Concept 2: Quality of life** | Quality of life, Life Quality, Health-Related Quality Of Life, Health Related Quality Of Life, HRQOL |

**Search strategy via Pubmed**

("inappropriate prescri*"[Title/Abstract] OR overdos*[Title/Abstract] OR over-dos*[Title/Abstract] OR underdos*[Title/Abstract] OR under-dos*[Title/Abstract] OR misdos*[Title/Abstract] OR mis-dos*[Title/Abstract] OR overprescri*[Title/Abstract] OR underprescri*[Title/Abstract] OR misprescri*[Title/Abstract] OR over-prescri*[Title/Abstract] OR under-prescri*[Title/Abstract] OR mis-prescri*[Title/Abstract] OR "inappropriate medic*"[Title/Abstract] OR "inappropriate drug*"[Title/Abstract] OR "inappropriate dosing"[Title/Abstract] OR "inappropriate therapy"[Title/Abstract] OR "inappropriate therapies"[Title/Abstract] OR "medication error"[Title/Abstract] OR "medication errors"[Title/Abstract] OR "medications error"[Title/Abstract] OR “medications errors”[Title/Abstract] OR “medicine errors”[Title/Abstract] OR “suboptimal medic*”[Title/Abstract] OR “suboptimal drug*”[Title/Abstract] OR "suboptimal prescri*"[Title/Abstract] OR "suboptimal dosing"[Title/Abstract] OR "suboptimal therapy"[Title/Abstract] OR "suboptimal therapies"[Title/Abstract] OR "inadequate medic*"[Title/Abstract] OR "inadequate drug*"[Title/Abstract] OR "inadequate prescri*"[Title/Abstract] OR "inadequate dosing"[Title/Abstract] OR "inadequate therapy"[Title/Abstract] OR "inadequate therapies"[Title/Abstract] OR "appropriate prescri*"[Title/Abstract] OR "prescription pattern"[Title/Abstract] OR "prescription patterns"[Title/Abstract] OR "prescriptions patterns"[Title/Abstract] OR "prescriptions pattern"[Title/Abstract] OR "prescribing pattern"[Title/Abstract] OR "prescribing patterns"[Title/Abstract] OR "high-risk medic*"[Title/Abstract] OR "inappropriate use"[Title/Abstract] OR "medication appropriateness"[Title/Abstract] OR "drug appropriateness"[Title/Abstract] OR "pharmacological inappropriateness"[Title/Abstract] OR "drug therapy problem"[Title/Abstract] OR "drug therapy problems"[Title/Abstract] OR ((inappropriate[Title/Abstract] OR inadequate[Title/Abstract] OR suboptimal[Title/Abstract]) AND “drug therapy”[Title/Abstract]) OR "harmful medic*"[Title/Abstract] OR "prescription appropriateness"[Title/Abstract] OR "prescribing appropriateness"[Title/Abstract] OR "prescription error"[Title/Abstract] OR "prescriptions errors"[Title/Abstract] OR "prescription errors"[Title/Abstract] OR "prescribing error"[Title/Abstract] OR "prescribing errors"[Title/Abstract] OR "medication safety"[Title/Abstract] OR "medications safety"[Title/Abstract] OR "drug safety"[Title/Abstract] OR "drugs safety"[Title/Abstract] OR "medicine safety"[Title/Abstract] OR "medicines safety"[Title/Abstract] OR "safe prescri*"[Title/Abstract] OR ("prescri*”[Title/Abstract] AND “drug”[Title/Abstract] AND (“misuse"[Title/Abstract] OR “overuse”[Title/Abstract] OR “underuse”[Title/Abstract])) OR ("prescri*”[Title/Abstract] AND “medic*”[Title/Abstract] AND (“misuse"[Title/Abstract] OR “overuse”[Title/Abstract] OR “underuse”[Title/Abstract])) OR “drug overdose”[Title/Abstract] OR "potentially inappropriate medication list"[Title/Abstract] OR “deprescri*”[Title/Abstract] OR “unnecessary prescri*”[Title/Abstract] OR (“potential prescri*”[Title/Abstract] AND “omissions”[Title/Abstract]) OR “irrational use of medic*”[Title/Abstract] OR “irrational use of drugs”[Title/Abstract] OR “prescribing cascade”[Title/Abstract] OR “prescription cascade”[Title/Abstract] OR “inappropriate polypharmacy”[Title/Abstract] OR “aberrant prescri*”[Title/Abstract] OR “quality of prescri*”[Title/Abstract] OR “Inappropriate prescribing”[MeSH Terms] OR “medication errors”[MeSH Terms] OR “prescription drug misuse”[MeSH Terms] OR “prescription drug overuse”[MeSH Terms] OR “drug overdose”[MeSH Terms] OR “potentially inappropriate medication list”[MeSH Terms] OR “deprescriptions”[MeSH Terms])

AND

(“Quality Of Life”[Title/Abstract] OR "Life Quality"[Title/Abstract] OR "Health-Related Quality Of Life"[Title/Abstract] OR "Health Related Quality Of Life"[Title/Abstract] OR "HRQOL"[Title/Abstract] OR “Quality Of Life”[MeSH Terms])

- **1,810 studies**
- **After screening 35 studies**
