# Supplementary material for: Quality of prescribing and health-related quality of life in older adults: a narrative review with a special focus on patients with atrial fibrillation and multimorbidity
Source: Eur Geriatr Med. 2025 Mar 9;16(4):1137–61. doi: 10.1007/s41999-025-01175-2 (PMC12378476; doi:10.1007/s41999-025-01175-2)
Supplement: Supplementary file 3 — Supplementary file3 (DOCX 36 KB) [file 41999_2025_1175_MOESM3_ESM.docx]

**Quality of Prescribing and Health-Related Quality of Life in Older Adults: A Narrative Review with a Special Focus on Patients with Atrial Fibrillation and Multimorbidity**

European Geriatric Medicine

*Cheima Amrouch^1,2^, Deirdre A. Lane^3,4,5^, Amaia Calderón-Larrañaga^6,7^, Mirko Petrovic^1*^, Delphine De Smedt^2*^, on behalf of the AFFIRMO investigators^§^*

*1 Department of Internal Medicine and Paediatrics, Ghent University, Ghent, Belgium
2 Department of Public Health and Primary Care, Ghent University, Ghent, Belgium
3 Liverpool Centre for Cardiovascular Science, University of Liverpool, Liverpool John Moores University and Liverpool Heart & Chest Hospital, William Henry Duncan Building, L7 8TX, Liverpool, UK
4 Department of Cardiovascular and Metabolic Medicine, Institute of Life Course and Medical Sciences, University of Liverpool, Liverpool, UK
5 Department of Clinical Medicine, Aalborg University, Aalborg, Denmark
6* *Department of Neurobiology, Aging Research Center, Care Sciences and Society, Karolinska Institutet and Stockholm University, Stockholm, Sweden
7* *Stockholm Gerontology Research Center, Stockholm, Sweden
*Shared last author
^§^List of coauthors in Appendix 1*

[*cheima.amrouch@ugent.be*](mailto:cheima.amrouch@ugent.be)

## **Supplementary tables**

Table S1. Examples of generic, population specific and disease specific health-related quality of life (HRQOL) and QOL tools reported in the included studies.

| **Generic HRQOL assessment tools** | **Characteristics** |
| --- | --- |
| EuroQol‐5D (EQ-5D)[1] | The EQ-5D tool comprises five dimensions: Mobility, Usual Activities, Self-care, Pain & Discomfort, and Anxiety & Depression. Each dimension is assessed across three, and more recently, five levels of severity. From this, an EQ-5D profile can be generated, and an EQ-5D index or utility score can be calculated to quantify overall health outcomes. |
| EuroQol- Visual Analog Scale (EQ-VAS)[1] | EQ-VAS is a visual scale used to measure the respondent’s overall perception of their health, ranging from 0 (worst health imaginable) to 100 (best health imaginable). |
| 36 and 12‐item Short‐Form Health Survey (SF-36/12)[2] | The instrument consists of either 36 items or 12 items, categorised into eight health profiles: Physical Functioning, Role-Physical, Bodily Pain, General Health, Vitality, Social Functioning, Role-Emotional, and Mental Health. From these profiles, two summary scores can be derived: the Physical Component Summary (PCS) and the Mental Component Summary (MCS**)**, providing an overall measure of physical and mental health outcomes. |
| 15-dimensional (15D) tool [3] | This instrument consists of 15 dimensions, each assessed across 5 levels, and can be used to generate both a profile and a single index score. The dimensions include: Breathing, Mental Function, Speech, Vision, Mobility, Usual Activities, Vitality, Hearing, Eating, Elimination, Sleeping, Distress, Discomfort and Symptoms, Sexual Activity, and Depression. |
| **Disease specific HRQOL assessment tools** | |
| Atrial Fibrillation Effect on Quality-of-Life (AFEQT)[4] | This instrument consists of four domains with a total of 20 items: Symptoms, Daily Activities, Treatment Concern, and Treatment Satisfaction. Both individual domain scores and a composite score can be calculated. |
| Minnesota Living with Heart Failure Questionnaire (MLHFQ)[5] | This instrument has a heterogeneous internal structure and consists of 21 items, organised into two or three dimensions: Physical, Emotional, and Social Environment. Both a total score and dimension-specific scores can be calculated. |
| **Broad QOL assessment tools** | |
| Life-threatening diseases (McGill QOL)[6] | This instrument consists of four subscales: Physical Symptoms, Psychological Symptoms, Outlook on Life, and Meaningful Existence. Each subscale focuses on a different aspect of well-being. |
| Dementia specific HRQOL (QUALIDEM)[7] | This tool is professionally rated, rather than self-reported by the patient, and consists of 49 items that describe observable behaviour. It is designed to assess specific behaviours based on direct observation, providing a structured evaluation from a clinician or professional’s perspective. |
| Patient-Reported Outcome Based Evaluation of Quality of Life (PROBE)[8] | The PROBE is a 6-question measure developed from key domains that impact the quality of life in patients with Crohn’s disease and ulcerative colitis, including Anxiety, Depression, Fatigue, Social satisfaction, and Pain. It was designed specifically for individuals with inflammatory bowel disease. |
| CASP-19/12 [9] | This instrument has been developed specifically for adults aged 60 years and over. It includes four dimensions: Control, Autonomy, Self-realisation, and Pleasure, with a total of 19 items (CASP-19). A shorter version, CASP-12, combines Control and Autonomy into one dimension, resulting in three dimensions and 12 items. Other variations with different numbers of items and dimensions are also used to assess well-being and quality of life. |

Table S2. Examples of medication screening tools.

| **Medication screening tool** | **Characteristics** |
| --- | --- |
| **Explicit tools** | |
| Screening Tool of Older Persons’ Prescriptions/Screening Tool to Alert to Right Treatment STOPP/START [10] | The STOPP criteria identify potential inappropriate medication (PIM) use, while the START criteria identify potential prescribing omissions (PPOs). The most recent version contains 133 STOPP criteria and 57 START criteria. This tool can be considered a mixed tool, as it combines both explicit and implicit characteristics in its approach to evaluating medication appropriateness. |
| American Geriatric Society (AGS) Beers [11] | The Beers Criteria consists of five lists: PIMs, PIMS due do drug-drug interactions, PIMS due to drug – disease or drug-syndrome interactions, Medications to be used with caution, and Drugs to avoid or adjust based on kidney function. Although the Beers Criteria is tailored for use in United Sates, it can also be applied internationally. |
| Screening Tool of Older Persons Prescriptions in older adults with high fall risk (STOPPFall)[12] | STOPPFall consists of 14 medication classes that are associated with an increased risk of falls. It is derived from the STOPP/START criteria. |
| **Implicit tools** | |
| Medication appropriateness index (MAI)[13] | This tool consists of 10 criteria or questions designed to assess the appropriateness of the prescribed medications. The criteria focus on several key aspects, including effectiveness, directions, therapeutic duplication and cost. |
| Assessing Quality of Care of Elderly (ACOVE)[14] | This tool consists of quality indicators (QI), explicitly phrased as IF-THEN clinical rules. The most recent version (ACOVE-3), includes 392 Qis that assess four domains of care: Screening and prevention, Diagnosis, Treatment, and Follow-up and continuity. ACOVE is designed to identify PIMs and PPOs. |

## **References**

1. Devlin, N., D. Parkin, and B. Janssen, *An Introduction to EQ-5D Instruments and Their Applications*, in *Methods for Analysing and Reporting EQ-5D Data*, N. Devlin, D. Parkin, and B. Janssen, Editors. 2020, Springer International Publishing: Cham. p. 1-22.

2. Ware, J., Jr., M. Kosinski, and S.D. Keller, *A 12-Item Short-Form Health Survey: construction of scales and preliminary tests of reliability and validity.* Med Care, 1996. **34**(3): p. 220-33.

3. Sintonen, H., *The 15D instrument of health-related quality of life: properties and applications.* Ann Med, 2001. **33**(5): p. 328-36.

4. Spertus, J., et al., *Development and validation of the Atrial Fibrillation Effect on QualiTy-of-Life (AFEQT) Questionnaire in patients with atrial fibrillation.* Circ Arrhythm Electrophysiol, 2011. **4**(1): p. 15-25.

5. Garin, O., et al., *Evidence on the global measurement model of the Minnesota Living with Heart Failure Questionnaire.* Qual Life Res, 2013. **22**(10): p. 2675-84.

6. Cohen, S.R., et al., *The Mcgill Quality-of-Life Questionnaire - a Measure of Quality-of-Life Appropriate for People with Advanced Disease - a Preliminary-Study of Validity and Acceptability.* Palliative Medicine, 1995. **9**(3): p. 207-219.

7. Ettema, T.P., et al., *QUALIDEM: development and evaluation of a dementia specific quality of life instrument. Scalability, reliability and internal structure.* Int J Geriatr Psychiatry, 2007. **22**(6): p. 549-56.

8. Barnes, E.L., et al., *A Novel Patient-Reported Outcome-Based Evaluation (PROBE) of Quality of Life in Patients With Inflammatory Bowel Disease.* Am J Gastroenterol, 2019. **114**(4): p. 640-647.

9. Frias-Goytia, G.L., et al., *A systematic review of quality of life (QoL) studies using the CASP scale in older adults.* Quality of Life Research, 2024.

10. O'Mahony, D., et al., *STOPP/START criteria for potentially inappropriate prescribing in older people: version 3.* Eur Geriatr Med, 2023. **14**(4): p. 625-632.

11. By the American Geriatrics Society Beers Criteria Update Expert, P., *American Geriatrics Society 2023 updated AGS Beers Criteria(R) for potentially inappropriate medication use in older adults.* J Am Geriatr Soc, 2023. **71**(7): p. 2052-2081.

12. Seppala, L.J., et al., *STOPPFall (Screening Tool of Older Persons Prescriptions in older adults with high fall risk): a Delphi study by the EuGMS Task and Finish Group on Fall-Risk-Increasing Drugs.* Age Ageing, 2021. **50**(4): p. 1189-1199.

13. Hanlon, J.T. and K.E. Schmader, *The Medication Appropriateness Index: A Clinimetric Measure.* Psychother Psychosom, 2022. **91**(2): p. 78-83.

14. Wenger, N.S., et al., *Introduction to the assessing care of vulnerable elders-3 quality indicator measurement set.* J Am Geriatr Soc, 2007. **55 Suppl 2**: p. S247-52.
